# Editorial: Biomechanics in Translation: From Vascular Biology to Cardiovascular Drug Discovery

**DOI:** 10.3389/fbioe.2020.00902

**Published:** 2020-08-19

**Authors:** Zhiyong Li, Suowen Xu

**Affiliations:** ^1^School of Biological Science and Medical Engineering, Southeast University, Nanjing, China; ^2^School of Mechanical, Medical and Process Engineering, Queensland University of Technology, Brisbane, QLD, Australia; ^3^Division of Life Sciences and Medicine, Department of Endocrinology and Metabolism, The First Affiliated Hospital, University of Science and Technology of China, Hefei, China

**Keywords:** biomechanics, atherosclerosis, vascular biology, cardiovascular disease, hemodynamics, mechanobiology

The Research Topic on “Biomechanics in Translation: From Vascular Biology to Cardiovascular Drug Discovery” collects novel studies of the cross-disciplinary research in biomechanics and vascular biology. This collection covers a wide range of research work from basic biology, computational modeling, material characterization, imaging to clinical studies. It provides a comprehensive overview of the advances in biomechanical technologies and their applications in diagnosis and treatment of cardiovascular diseases (CVD).

CVD is a major health burden and the leading cause of mortality and morbidity worldwide. The main underlying cause of CVD is atherosclerosis, where abnormal deposits of fat, cholesterol, and other substances build up in the inner lining of the arteries to form plaque. Plaques often rupture without warning, causing acute vascular events, such as myocardial infarction and stroke, which are life-threatening events that occur when a blood vessel supplying the heart or brain is suddenly blocked, causing damage to the tissue and its function. A better understanding of plaque morphology, mechanical behavior, and hemodynamics is essential for the identification of vulnerable plaques pre-rupture. This Research Topic collected three studies covering these three important aspects. To better understand the mechanical response of plaque tissue under cyclic loading conditions, Paritala et al. developed a methodology to experimentally and numerically characterize the stress-relaxation and cyclic mechanical behavior of carotid plaque tissues. The time-dependent mechanical response of the carotid plaque tissue was investigated by applying cyclic loading under physiological temperature and hydration medium. There was a decrease in the peak force as a function of cycle number indicating mechanical distension due to repeated loading. The tissue accumulated residual deformation as a function of cycle number, which was attributed to fatigue softening. This work represents a step toward an improved understanding of the material behavior of the human atherosclerotic plaques, which may assist in plaque vulnerability assessment.

To better understand the impact of hemodynamics on plaque stability, Wang et al. combined *in vivo* MR imaging, histological analysis, and computational simulation to investigate the hemodynamics in a patient with a tandem carotid stenosis. In this study, a patient with a tandem carotid stenosis at both internal carotid artery and common carotid artery was recruited. Multi-sequence pre-surgery MR imaging and post-surgery carotid plaque tissue samples were collected for analysis. The 3D patient-specific geometrical model was reconstructed from the *in vivo* MR imaging. Computational fluid dynamics (CFD) simulation was conducted to investigate how the tandem stenosis developed and affected each other. The results of this study suggest that when planning carotid endarterectomy, CFD simulation on the presumptive models could help clinicians to estimate the blood flow behavior after surgery. Particular attention should be paid to the case of tandem stenosis, as the local hemodynamic environment is more complex and treatment of one stenosis may lead to a variation in the hemodynamic loading on the second plaque, which may result in either a higher risk of plaque rupture or restenosis.

In terms of plaque morphology, He C. et al. developed a machine learning algorithm to segment plaque components from *in vivo* optical coherence tomography (OCT) imaging. Intravascular OCT has been used to characterize coronary plaque morphology in recent years. There is an urgent need to develop a validated algorithm for plaque characterization which can help facilitate the standardization of OCT image interpretation of plaque morphology. In He C. et al.'s study, a total of 31 patients underwent carotid endarterectomy and the *ex vivo* carotid plaques were imaged with OCT. Optical parameter, texture features and relative position of pixels were extracted and then used to quantify the tissue characterization of plaque components. The results show the developed algorithm was capable of characterizing plaque components with an excellent accuracy using the combined feature set.

Vascular calcification is an abnormal cell-mediated process in which bone-specific hydroxyapatite crystals are actively deposited on the blood vessel wall and is a significant pathological basis for the increased incidence and mortality of adverse cardiovascular events. Macrophages play an important regulatory role in the occurrence, development, and regression of vascular calcification. Li et al. summarizes a wealth of research in this field and explores the roles of macrophages in the development process of vascular calcification. It was pointed out that further exploration of the potential molecular mechanism of how macrophages regulate the progression and regression of vascular calcification is expected to reveal a new entry point for the prevention and treatment of vascular calcification, with far-reaching implications.

This collection also includes applications of biomechanics in design or evaluation of medical devices such as stents and valves. Coronary artery stenting is commonly used for the treatment of coronary stenosis, and different stent structures have various impacts on the stress distribution within the plaque and artery as well as the local hemodynamic environment. To better understand the mechanical interaction between blood flow, stent, and artery, Wei et al. performed both structural and hemodynamic analyses to evaluate the performance of different stent structures. Structural analyses were firstly performed to obtain the deformed luminal boundaries of a curved artery. Hemodynamic analyses were then conducted to quantify the critical hemodynamic parameters. The effects of stent structure by changing the structure and connection way of the stent struts were investigated. This study provides a better understanding of the deployment of different stents inside curved stenotic arteries to facilitate the clinical choice of suitable commercial stents. In another study, Zhu et al. used biomechanics to evaluate the pulmonary flow conditions. Recent developments in the expanded-polytetrafluoroethylene (ePTFE) pulmonary prosthetic valves provide promising progress in the management procedures of various congenital heart diseases. In Zhu et al.'s study, the hemodynamic characteristics of bileaflet and trileaflet ePTFE valve designs were experimentally evaluated under pulmonary flow conditions. The results indicate that the current bileaflet prosthetic valve design is capable of providing a better overall hemodynamic performance than the trileaflet design. In a clinical study, He D. et al. developed a new approach of 3D-printed extravascular stenting of the left renal vein for the treatment of nutcracker syndrome (NCS). 28 patients were admitted for laparoscopic 3D-printed extravascular stenting treatment. After treatment, the NCS symptoms in all patients resolved or improved during the follow-up period. Compared to endovascular stenting or polytetrafluoroethylene artificial vessel procedures, 3D-printed polyetheretherketone extravascular stenting has more advantages in terms of stent design and rigidity and approach rationality while successfully preventing stent migration and thrombosis. The method may serve as an accurate and effective treatment for NCS patients.

This exciting collection of papers represent the recent developments in biomechanics and vascular biology with state-of-the-art contributions and critical reviews on the new advances in this growing field, with an emphasis on the interface between engineering and clinical medicine. We are delighted to be a part of this effort and witness the rapid expansion of the biomechanics in clinical applications, providing more opportunities for future discussions and exchanges of ideas and experiences in translating biomechanics into a wider range of applications.

## Author Contributions

All authors listed have made a substantial, direct and intellectual contribution to the work, and approved it for publication.

## Conflict of Interest

The authors declare that the research was conducted in the absence of any commercial or financial relationships that could be construed as a potential conflict of interest.

